# Supplementary material for: Disentangling the Janus-faced effects of cations in electrocatalysis
Source: Nat Commun. 2026 Mar 31;17:3149. doi: 10.1038/s41467-026-71126-3 (PMC13043930; doi:10.1038/s41467-026-71126-3)
Supplement: Supplementary file 1 — Supplementary Information [file 41467_2026_71126_MOESM1_ESM.pdf]

Supplementary Information

Disentangling the Janus-faced effects of cations in electrocatalysis

Xinwei Zhu[1,2], Tobias Binninger[1], Marc T.M. Koper[3], Michael Eikerling[1,2]

[1] *Theory and Computation of Energy Materials (IET-3), Institute of Energy Technologies, Forschungszentrum Jülich GmbH, 52425 Jülich, Germany*

[2] *Chair of Theory and Computation of Energy Materials, Faculty of Georesources and Materials Engineering, RWTH Aachen University, 52062 Aachen, Germany*

[3] *Leiden Institute of Chemistry, Leiden University, 2333CC Leiden, The Netherlands*

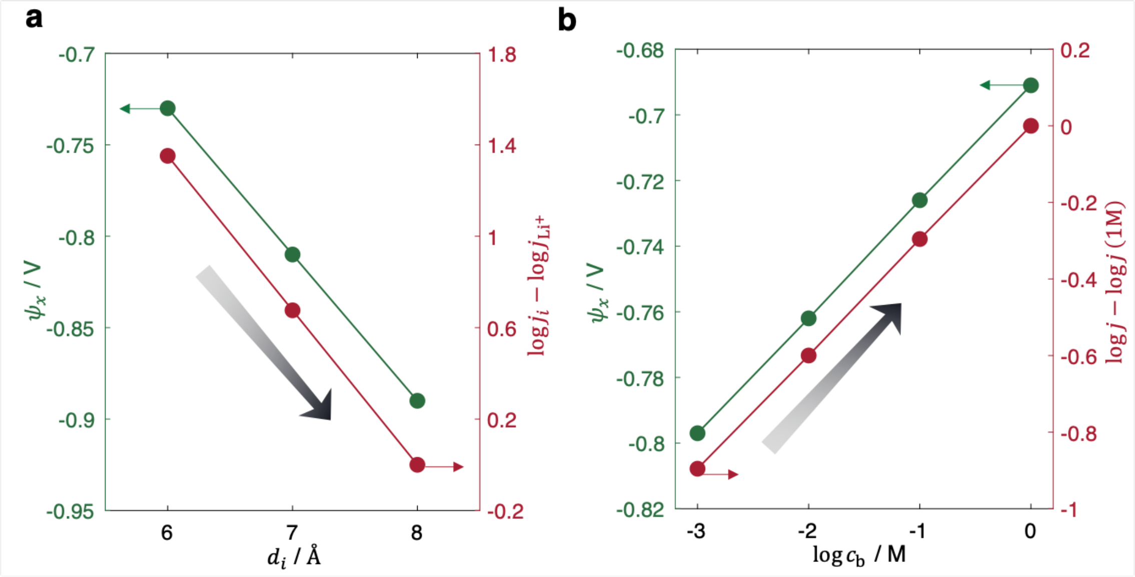

Fig. S1: Cation effects from the view of Frumkin corrections assuming the reaction plane lies at the IHP. (a) Dependence of ψ_x (assumed $\psi_x = \psi_{IHP}$) and HER rate on effective cation size, using $c_b = 0.1$ M. (b) Dependence of ψ_x and HER rate on supporting cation concentration, using $d_i = 6$ Å. The results are calculated using Eqs. 1 and 2. α is set to the typical value of 0.5. $E_M = -0.6$ V versus the reversible hydrogen electrode (RHE) and pH = 13. Other parameters are given in Table 1.

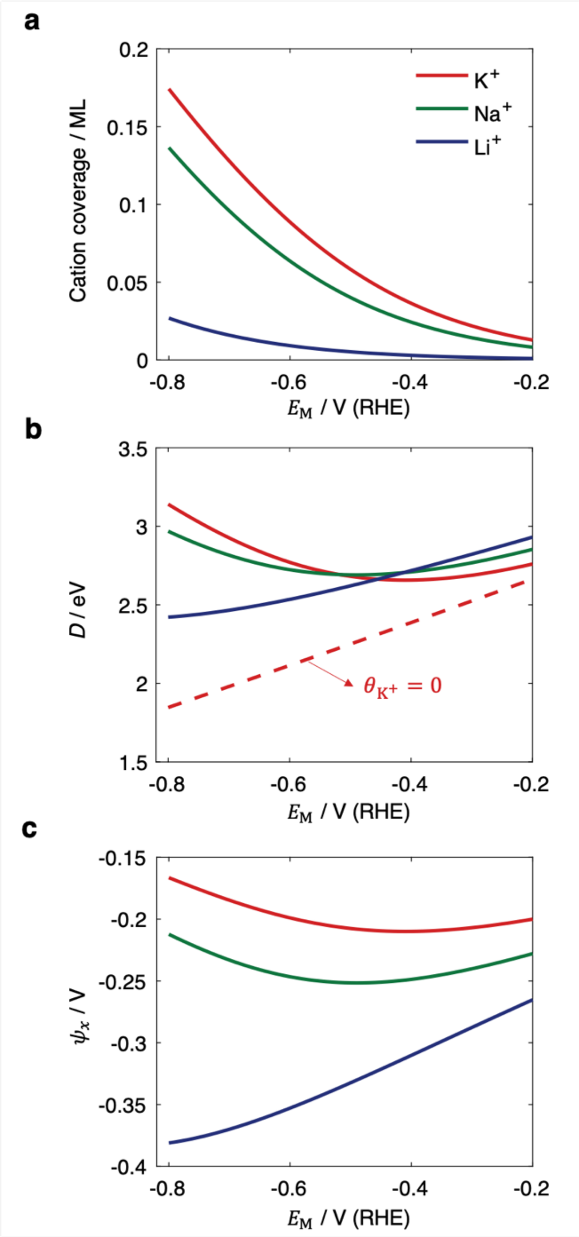

Fig. S2: Cation identity effects on interfacial properties. (a) Simulated cation coverages on Au in 0.1 M KOH, NaOH, and LiOH. (b) Simulated H-OH bond strength as the function of applied potential. The result for K^+ without considering cation adsorption is shown for comparison. (c) Simulated ψ_x (assumed $\psi_x = \psi_{\mathrm{OHP}}$) as the function of applied potential. Parameters used are given in Table 1.

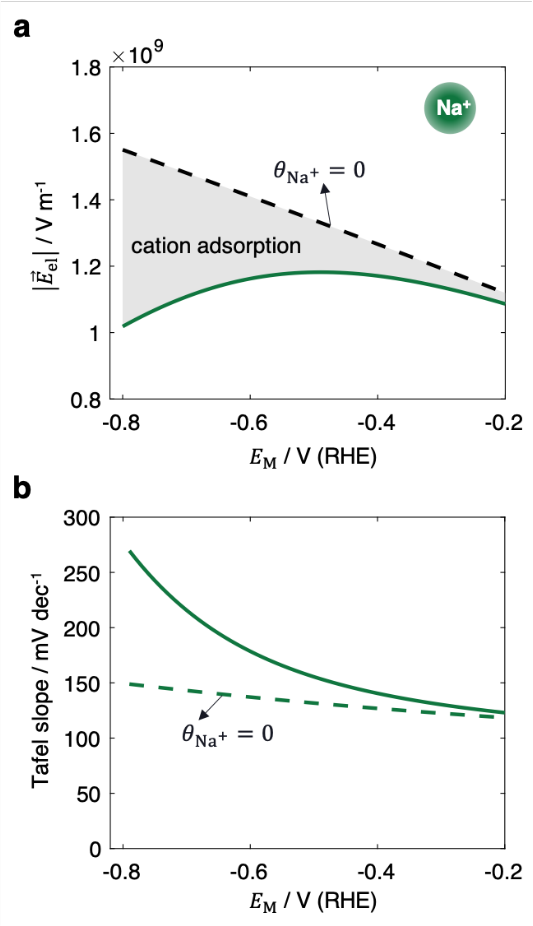

Fig. S3: Influence of Na^+ adsorption on the interfacial electric field and HER kinetics. (a) Calculated electric field strength within the OHL for Na^+. (b) Influence of Na^+ adsorption on the Tafel slope. Parameters used are given in Table 1.

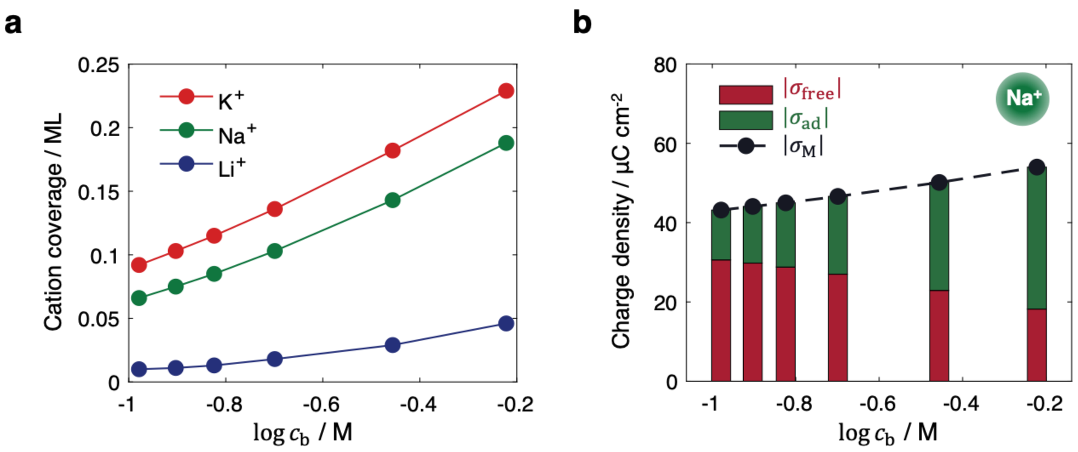

Fig. S4: Cation concentration effects on the adsorption and surface charge densities. (a) Influence of cation concentration on cation adsorption coverages. (b) Influence of cation concentration on surface charge densities for Na^+. $E_{\mathrm{M}} = -0.6$ V versus RHE and pH = 13. The results are simulated using Eqs. 3-8 and parameters given in Table 1.

Supplementary Note 1: Parameter sensitivity analysis

The sensitivity of the model results to the parameters ξ_i, γ_i, ϵ_1 and ϵ_2 is examined by independently varying each parameter while keeping the effective size $d_i = 6$ Å and the equilibrium adsorption potential $E_i^0 = -1.19$ V fixed. All remaining parameters are set to the values listed in Table 1. The impact of these variations on key observables, including the cation adsorption coverage, the EDL characteristics (ψ_x and $|\vec{E}_{\mathrm{el}}|$), and the HER current density is systematically analyzed.

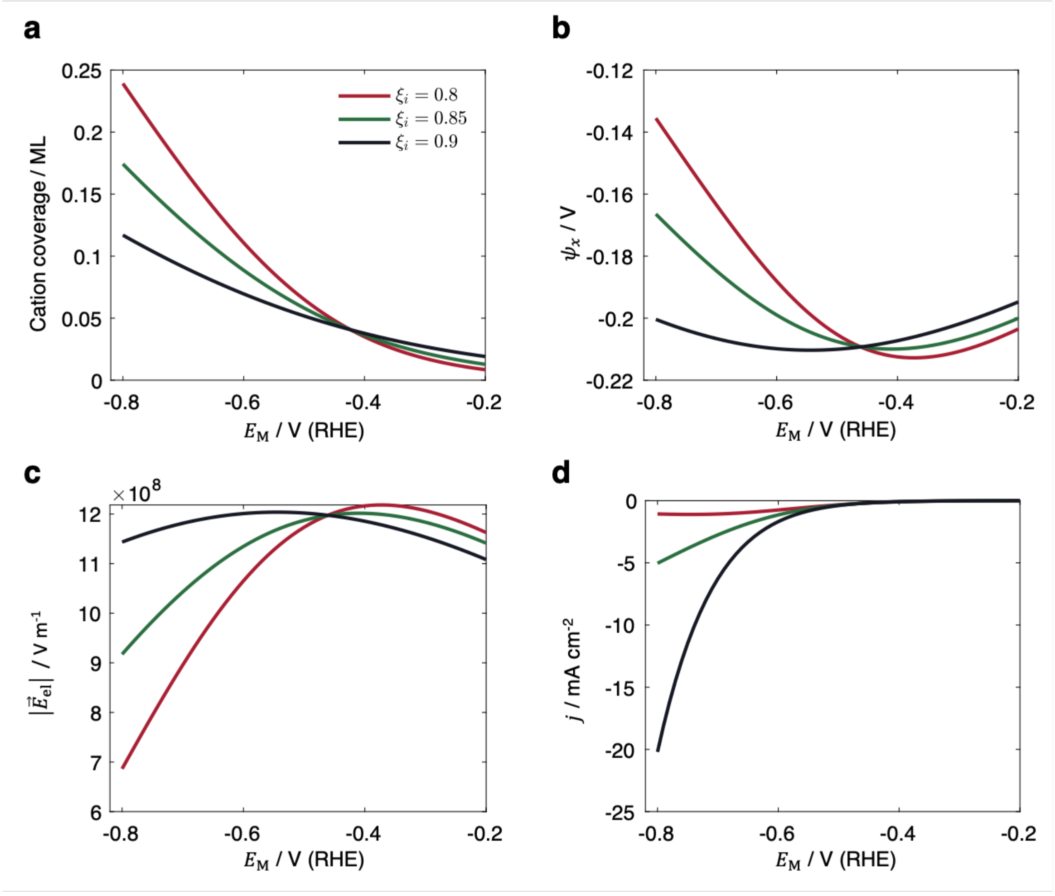

Fig. S5: Sensitivity analysis of ξ_i. Influence of ξ_i on (a) Cation coverage, (b) ψ_x (assumed $\psi_x = \psi_{\mathrm{OHP}}$), (c) $|\vec{E}_{\mathrm{el}}|$, and (d) HER current density. Conditions: $c_{\mathrm{b}} = 0.1$ M and pH = 13.

ξ_i influences the cation adsorption isotherm through Eq. 4. A smaller ξ_i corresponds to a larger degree of partial charge transfer, leading to a steeper increase in cation adsorption with increasingly negative potential, as shown in Fig. S5a. In addition, ξ_i affects the EDL characteristics through the surface charge contribution $\sigma_{\mathrm{ad}} = e_0\rho\xi_i\theta_i$ in Eq. 3, which modifies both the local potential and the electric field strength, as illustrates in Fig. S5b and S5c. While

variations in ξ_i rescale the magnitude of the HER current density, they do not reproduce the experimentally observed inversion of cation trends, Fig. S5d.

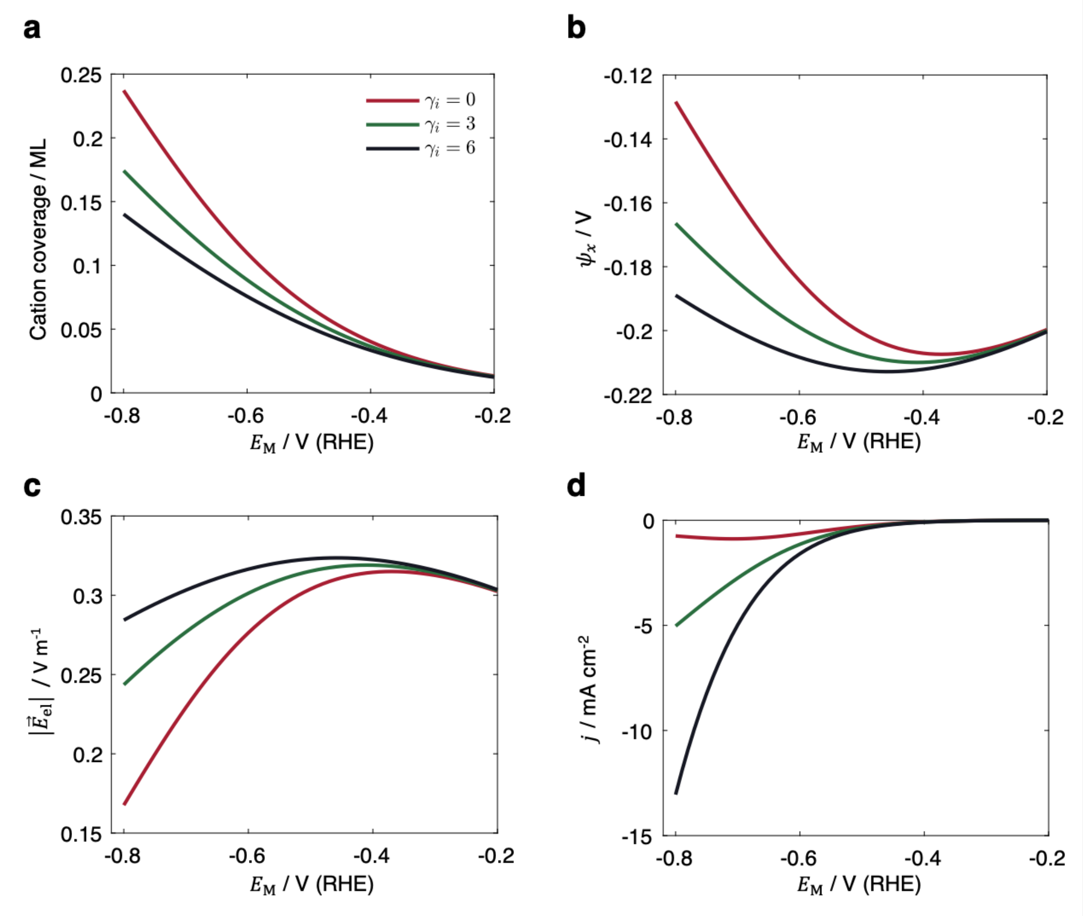

Fig. S6: Sensitivity analysis of γ_i. Influence of γ_i on (a) Cation coverage, (b) ψ_x (assumed $\psi_x = \psi_{OHP}$), (c) $|\vec{E}_{el}|$, and (d) HER current density. Conditions: $c_b = 0.1$ M and pH = 13.

γ_i represents the lateral repulsion between adsorbed cations in the Frumkin isotherm in Eq. 4. A larger γ_i increases the effective repulsive interaction, thereby inhibiting cation adsorption, as shown in Fig. S6a. As a consequence, ψ_x is shifted to more negative values, while $|\vec{E}_{el}|$ is enhanced with increasing γ_i, as shown in Fig. S6b and S6c. The competition between these two effects leads to an overall increase in the HER current density with increasing γ_i, Fig. S6d.

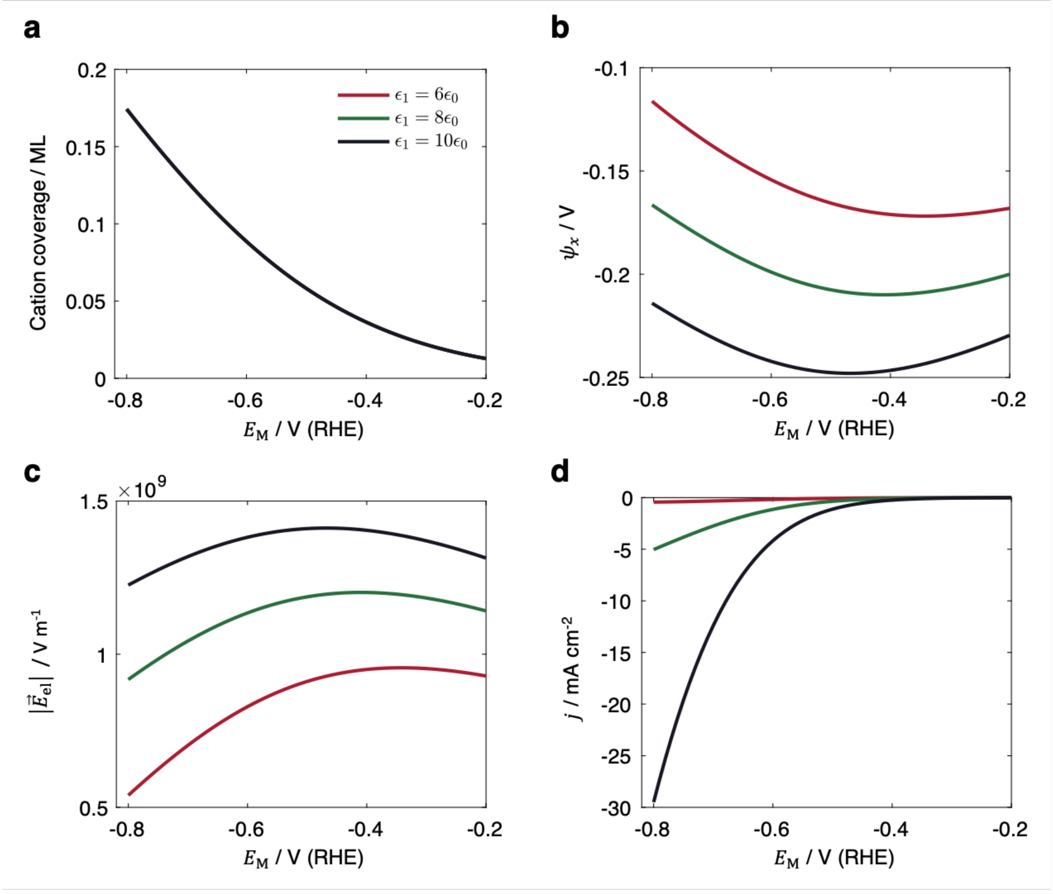

Fig. S7: Sensitivity analysis of ϵ_1. Influence of ϵ_1 on (a) Cation coverage, (b) ψ_x (assumed $\psi_x = \psi_{\mathrm{OHP}}$), (c) $|\vec{E}_{\mathrm{el}}|$, and (d) HER current density. Conditions: $c_{\mathrm{b}} = 0.1$ M and pH = 13.

The interfacial dielectric constant ϵ_1 has no influence cation adsorption, as it does not enter the adsorption isotherm in Eq. 4. Increasing ϵ_1 shifts ψ_x to more negative values, while enhances $|\vec{E}_{\mathrm{el}}|$. The combined effect leads to an overall increase in the HER current density.

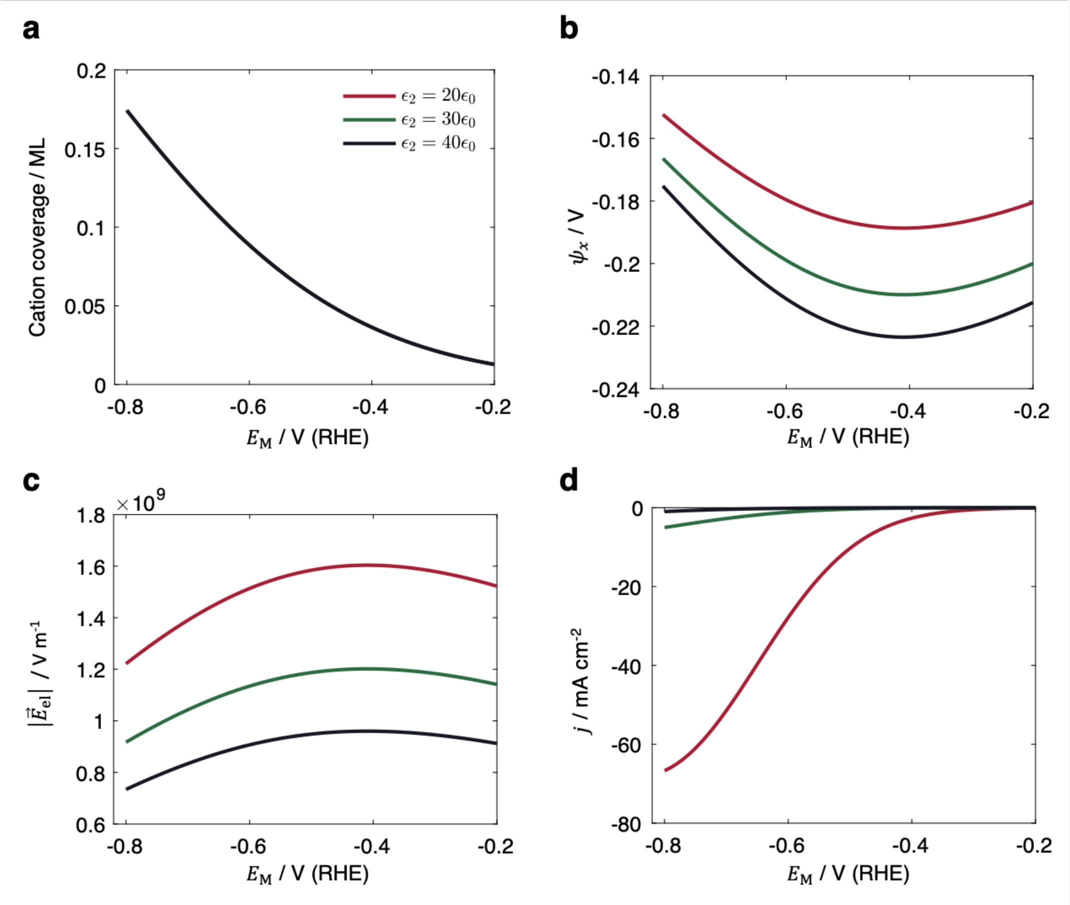

Fig. S8: Sensitivity analysis of ϵ_2. Influence of ϵ_2 on (a) Cation coverage, (b) ψ_x (assumed $\psi_x = \psi_{OHP}$), (c) $|\vec{E}_{el}|$, and (d) HER current density. Conditions: $c_b = 0.1$ M and pH = 13.

Similar to ϵ_1, ϵ_2 has no influence on cation adsorption. And increasing ϵ_2 shifts ψ_x to more negative values. In contrast to the effect of ϵ_1, however, the local electric field $|\vec{E}_{el}|$ is weakened with increasing ϵ_2, because the electric field strength is proportional to $|\sigma_{free}|/\epsilon_2$. The combined effect leads to an overall decrease in the HER current density.

Supplementary Note 2: MATLAB codes to reproduce figures

Fig. 2b

```
%% Parameters

clc
clear all

% experimental conditions
T  = 298; % [K] temperature
pH = 13; % solution pH
phiM = -0.6; % applied potential vs RHE
cC0 = 0.1; % [M] bulk cation concentration

% constant parameters
NA = 6.02e23; % [1/mol] Avogadro number
e0 = 1.6e-19; %[C] elementary charge
kB = 1.38e-23; %[J/K] Boltzmann constant
F = e0*NA; % [C/mol] Faraday constant
R = kB*NA; % [J/mol/K] gas constant
eps0 = 8.85e-12; %[F/m] vacuum permittivity

% EDL parameters
Nad = 1.4e19; % [m^-2] number density of active sites
epsb = 78.5*eps0; % [F/m] bulk permittivity
delta1 = 2.0e-10; % [m] thickness of Inner Helmholtz layer (IHL)
eps1 = 8*eps0; % [F/m] permittivity of the IHL
eps2 = 30*eps0; % [F/m] permittivity of the OHL
lD = sqrt(epsb*R*T/F^2/2/(cC0*1e3)); % [m] Debye length
phipzc = 0.5; % [V] pzc vs SHE

% kinetic parameter
alpha = 0.5; % transfer coefficient

%% Solve EDL model

dion_list = [6e-10 7e-10 8e-10]; % [m] effective cation size list

for k_size = 1:length(dion_list)

   dion = dion_list(k_size); % [m] effective cation size
   delta2 = dion/2; % [m] thickness of outer Helmholtz layer (OHL)
   fsize = 2*cC0*1e3*(dion)^3*NA; % size factor

   EDL =  @(x)sign(x)*2*R*T/F*asinh(sqrt(1/2/fsize*(exp(fsize/2*(F*lD*x/R/T/epsb)^2)-1))) +
x*(delta1/eps1+delta2/eps2)...
      - (phiM - 0.059*pH - phipzc); % Eq.2
   x0 = [-0.1]; % initial guess
   lb = [-1]; % lower limit
   ub = [1]; % upper limit
   Res=lsqnonlin(EDL,x0,lb,ub); % nonlinear optimization

   sigmafree(k_size) = Res(1); % [C/m^2] free surface charge density
```

```
   phi_OHP(k_size) =
sign(sigmafree(k_size))*2*R*T/F*asinh(sqrt(1/2/fsize*(exp(fsize/2*(F*lD*sigmafree(k_size)/R/T/epsb)^2)-
1))); % [V] OHP potential
   phi_IHP(k_size) = phi_OHP(k_size) + sigmafree(k_size)/eps2*delta2; % [V] IHP potential

end

%% Plot the results

color1 = [42 110 63]/255;
color2 = [168 30 50]/255;

dlogj = alpha*F/R/T/log(10)*(phi_OHP-phi_OHP(end)); % Eq 1, taking dion = 8e-10 as reference

figure
hold on;box on

yyaxis left
plot(dion_list*1e10,phi_OHP,'-o','LineWidth',1,'color',color1,'MarkerEdgeColor',
color1,'MarkerFaceColor',color1,'MarkerSize', 8);
ylim([-0.4 -0.26])
set(gca, 'YColor', color1)

yyaxis right
plot(dion_list*1e10,dlogj,'-o','LineWidth',1,'color',color2,'MarkerEdgeColor',
color2,'MarkerFaceColor',color2,'MarkerSize', 8);
ylim([-0.2 1.0])
set(gca, 'YColor', color2)

set(gca,'XColor', [0 0 0],'FontSize', 12);
xlim([5.5 8.5])
xticks([6 7 8])
```

Fig. 2c

```
%% Parameters

clc
clear all

% experimental conditions
T  = 298; % [K] temperature
pH = 13; % solution pH
phiM = -0.6; % applied potential vs RHE

% constant parameters
NA = 6.02e23; % [1/mol] Avogadro number
e0 = 1.6e-19; %[C] elementary charge
kB = 1.38e-23; %[J/K] Boltzmann constant
F = e0*NA; % [C/mol] Faraday constant
R = kB*NA; % [J/mol/K] gas constant
eps0 = 8.85e-12; %[F/m] vacuum permittivity

% EDL parameters
Nad = 1.4e19; % [m^-2] number density of active sites
epsb = 78.5*eps0; % [F/m] bulk permittivity
delta1 = 2.0e-10; % [m] thickness of Inner Helmholtz layer (IHL)
eps1 = 8*eps0; % [F/m] permittivity of the IHL
dion = 6e-10; % [m] effective cation size
delta2 = dion/2; % [m] thickness of outer Helmholtz layer (OHL)
eps2 = 30*eps0; % [F/m] permittivity of the OHL
phipzc = 0.5; % [V] pzc vs SHE

% kinetic parameter
alpha = 0.5; % transfer coefficient

%% solve the model

con_list = [0.001 0.01 0.1 1]; % [M] cation concentration list

for k_con = 1:length(con_list)

   cC0 = con_list(k_con); % [M] bulk cation concentration
   lD = sqrt(epsb*R*T/F^2/2/(cC0*1e3)); % [m] Debye length
   fsize = 2*cC0*1e3*(dion)^3*NA; % size factor

   EDL = @(x)sign(x)*2*R*T/F*asinh(sqrt(1/2/fsize*(exp(fsize/2*(F*lD*x/R/T/epsb)^2)-1))) + x*(delta1/eps1+delta2/eps2)...
      - (phiM - 0.059*pH - phipzc); % Eq. 2
   x0 = [-0.1]; % initial guess
   lb = [-1]; % lower limit
   ub = [1]; % upper limit
   Res=lsqnonlin(EDL,x0,lb,ub); % nonlinear optimization

   sigmafree(k_con) = Res(1); % [C/m^2] free surface charge density
   phi_OHP(k_con) = sign(sigmafree(k_con))*2*R*T/F*asinh(sqrt(1/2/fsize*(exp(fsize/2*(F*lD*sigmafree(k_con)/R/T/epsb)^2)-1))); % [V] OHP potential
   phi_IHP(k_con) = phi_OHP(k_con) + sigmafree(k_con)/eps2*delta2; % [V] IHP potential
```

```
end

%% plot the results

color1 = [42 110 63]/255;
color2 = [168 30 50]/255;

dlogj = alpha*F/R/T/log(10)*(phi_OHP-phi_OHP(end)); % Eq. 1, taking cC0 = 1M as reference

figure
hold on;box on

yyaxis left
plot(log10(con_list),phi_OHP,'-o','LineWidth',1,'color',color1,'MarkerEdgeColor',
color1,'MarkerFaceColor',color1,'MarkerSize', 8);
ylim([-0.4 -0.2])
set(gca, 'YColor', color1)

yyaxis right
plot(log10(con_list),dlogj,'-o','LineWidth',1,'color',color2,'MarkerEdgeColor',
color2,'MarkerFaceColor',color2,'MarkerSize', 8);
ylim([-1.4 0.2])
set(gca, 'YColor', color2)

set(gca,'XColor', [0 0 0],'FontSize', 12);
xlim([-3.1 0.1])
xticks([-3 -2 -1 0])
```

Fig. 4

```
%% Parameters

clc
clear all

% experimental conditions
T  = 298; % [K] temperature
pH = 13; % solution pH
phiM = -0.6; % applied potential vs RHE
cC0 = 0.1; % [M] bulk cation concentration

% constant parameters
NA = 6.02e23; % [1/mol] Avogadro number
e0 = 1.6e-19; %[C] elementary charge
kB = 1.38e-23; %[J/K] Boltzmann constant
F = e0*NA; % [C/mol] Faraday constant
R = kB*NA; % [J/mol/K] gas constant
eps0 = 8.85e-12; %[F/m] vacuum permittivity

% EDL parameters
Nad = 1.4e19; % [m^-2] number density of active sites
epsb = 78.5*eps0; % [F/m] bulk permittivity
delta1 = 2.0e-10; % [m] thickness of Inner Helmholtz layer (IHL)
eps1 = 8*eps0; % [F/m] permittivity of the IHL
dion = 6e-10; % [m] effective cation size
delta2 = dion/2; % [m] thickness of Outer Helmholtz layer (OHL)
eps2 = 30*eps0; % [F/m] permittivity of the OHL
lD = sqrt(epsb*R*T/F^2/2/(cC0*1e3)); % [m] Debye length
fsize = 2*cC0*1e3*(dion)^3*NA; % size factor
phipzc = 0.5; % [V] pzc vs SHE
Xi = 0.85; % [1] charge number per adsorbed cation

% kinetic parameter
alpha = 0.5; % transfer coefficient

%% Solve the model

theta_list = [0 0.05 0.1 0.15 0.2]; % [ML] cation adsorption coverage list

for k_theta = 1:length(theta_list)

   theta = theta_list(k_theta); % [ML] cation adsorption coverage
   sigma_ad = e0*Nad*Xi*theta; % surface charge contribution of adsorbed cations

   EDL =  @(x)sign(x)*2*R*T/F*asinh(sqrt(1/2/fsize*(exp(fsize/2*(F*lD*x/R/T/epsb)^2)-1))) +
x*(delta1/eps1+delta2/eps2)...
      - sigma_ad*delta1/eps1 -...
      (phiM - phipzc - 0.059*pH); % Eq. 3
   x0 = [-0.1]; % initial guess
   lb = [-1]; % lower limit
   ub = [1]; % upper limit
   Res=lsqnonlin(EDL,x0,lb,ub); % nonlinear optimization

   sigmafree(k_theta) = Res(1); % [C/m^2] free surface charge density
```

```
   phi_OHP(k_theta) =
sign(sigmafree(k_theta))*2*R*T/F*asinh(sqrt(1/2/fsize*(exp(fsize/2*(F*lD*sigmafree(k_theta)/R/T/epsb)^2)-
1))); % [V] OHP potential
   phi_IHP(k_theta) = phi_OHP(k_theta) + sigmafree(k_theta)/eps2*delta2; % [V] IHP potential
   E_OHL(k_theta) = -sigmafree(k_theta)/eps2; % [V/m] electric field strength, Eq. 5

end

color1 = [42 110 63]/255;
color2 = [168 30 50]/255;

dlogj = alpha*F/R/T/log(10)*(phi_OHP-phi_OHP(1)); % Eq. 1, taking theta = 0 as reference

figure(1) % Figure 4a
hold on;box on

yyaxis left
plot(theta_list,phi_OHP,'-o','LineWidth',1,'color',color1,'MarkerEdgeColor',
color1,'MarkerFaceColor',color1,'MarkerSize', 8);
ylim([-0.3 -0.1])
set(gca, 'YColor', color1)

yyaxis right
plot(theta_list,dlogj,'-o','LineWidth',1,'color',color2,'MarkerEdgeColor',
color2,'MarkerFaceColor',color2,'MarkerSize', 8);
ylim([-0.05 1.5])
set(gca, 'YColor', color2)

set(gca,'XColor', [0 0 0],'FontSize', 12);
xlim([-0.02 0.22])
xticks([0 0.05 0.1 0.15 0.2])

figure(2) % Figure 4c
hold on;box on

yyaxis left
plot(theta_list,-sigmafree*100,'-o','LineWidth',1,'color',color1,'MarkerEdgeColor',
color1,'MarkerFaceColor',color1,'MarkerSize', 8);
ylim([15 45])
set(gca, 'YColor', color1)

yyaxis right
plot(theta_list,E_OHL,'-o','LineWidth',1,'color',color2,'MarkerEdgeColor',
color2,'MarkerFaceColor',color2,'MarkerSize', 8);
ylim([0.4e9 1.6e9])
set(gca, 'YColor', color2)

set(gca,'XColor', [0 0 0],'FontSize', 12);
xlim([-0.02 0.22])
xticks([0 0.05 0.1 0.15 0.2])
```

Fig. 5, Fig. S2, and Fig. S3

```
%% Parameters

clc
clear all

% Experimental conditions
T  = 298; % [K] temperature
cC0 = 0.10; % [M] bulk cation concentration
pH = 13; % solution pH
phi_range_RHE = [-0.8:0.01:-0.2]; % [V] potential range vs RHE

% Constant parameters
NA = 6.02e23; % [1/mol] Avogadro number
e0 = 1.6e-19; %[C] elementary charge
h = 6.626e-34; % [J.s] Planck constant
kB = 1.38e-23; %[J/K] Boltzmann constant
F = e0*NA; % [C/mol] Faraday constant
R = kB*NA; % [J/mol/K] gas constant
eps0 = 8.85e-12; %[F/m] vacuum permittivity

% EDL parameters
epsb = 78.5*eps0; % [F/m] bulk permittivity
delta1 = 2.0e-10; % [m] thickness of Inner Helmholtz layer (IHL)
eps1 = 8*eps0; % [F/m] permittivity of the IHL
eps2 = 30*eps0; % [F/m] permittivity of the OHL
lD = sqrt(epsb*R*T/F^2/2/(cC0*1e3)); % [m] Debye length
phipzc = 0.5; % [V] pzc vs SHE
Nad = 1.4e19; % [m^-2] number density of active sites
Xi = 0.85; % [1] charge number per adsorbed cation
theta_max = 0.5; % maximum coverage
gamma = 3; % lateral interaction coefficient

% Kinetic parameters
lambda = 3; % [eV] reorganization energy
E_eq = -1.24; % [V] equilibrium potential of hydrogen adsorption
delta_el = 3; % [eV] electronic interaction strength
k0 = 1*kB*T/h; % [1/s] prefactor
B = 1.7e-9; % [e0.m] coefficient for electric field effects, Eq.6
D0 = 4.7; % [eV] field-free H-OH bond strength

%% Solve the model

dion_list = [6e-10 7e-10 8e-10]; % [m] effective cation size, [K, Na, Li]
Eref_list = [-1.19 -1.27 -1.65]; % [V] cation adsorption potential, [K, Na, Li]

for k_dion = 1:length(dion_list)

   dion = dion_list(k_dion); % [m] effective cation size
   delta2 = dion/2; % [m] thickness of outer Helmholtz layer (OHL)
   fsize = 2*cC0*1e3*(dion)^3*NA; % size factor
   E_ref = Eref_list(k_dion); % [V] cation adsorption potential

   for k_phi = 1:length(phi_range_RHE)
```

```
phiM = phi_range_RHE(k_phi); % [V] electrode potential vs RHE

% solving the cation adsorption coverage
Coverage = @(x)log(x/(theta_max-x))+ gamma*x + (1-Xi)*e0*(phiM-0.059*pH-E_ref)/kB/T - log(cC0); % Eq.4
x0 = [0.1]; % initial guess
lb = [0]; % lower limit
ub = [theta_max]; % upper limit
Res=lsqnonlin(Coverage,x0,lb,ub); % nonlinear optimization
theta(k_dion,k_phi) = Res(1); % cation adsorption coverage
sigma_ad(k_dion,k_phi) = e0*Nad*Xi*theta(k_dion,k_phi); % surface charge contribution of adsored cations

% solving the EDL model
EDL =  @(x)sign(x)*2*R*T/F*asinh(sqrt(1/2/fsize*(exp(fsize/2*(F*lD*x/R/T/epsb)^2)-1))) ...
   + x*(delta1/eps1+delta2/eps2)...
   - sigma_ad(k_dion,k_phi)*delta1/eps1 ...
   -(phiM - phipzc -0.059*pH); % Eq.3
x0 = [-0.1]; % initial guess
lb = [-10]; % lower limit
ub = [10]; % upper limit
Res=lsqnonlin(EDL,x0,lb,ub); % nonlinear optimization

sigmafree(k_dion,k_phi) = Res(1); % [C/m^2] free surface charge density
phi_OHP(k_dion,k_phi) = sign(sigmafree(k_dion,k_phi))*2*R*T/F*asinh(sqrt(1/2/fsize*(exp(fsize/2*(F*lD*sigmafree(k_dion,k_phi)/R/T/epsb)^2)-1))); % OHP potential
phi_IHP(k_dion,k_phi) = phi_OHP(k_dion,k_phi)+sigmafree(k_dion,k_phi)/eps2*delta2; % IHP potential
E_OHL(k_dion,k_phi) = -sigmafree(k_dion,k_phi)/eps2; % [V/m] electric field strength, Eq.5
delta_G(k_dion,k_phi) = phiM - 0.059*pH - E_eq - phi_OHP(k_dion,k_phi); % [eV] changes of Gibbs free energy
D(k_dion,k_phi) = -B*E_OHL(k_dion,k_phi) + D0; % [eV] bond strength, Eq.6
Ga(k_dion,k_phi) = (lambda+D(k_dion,k_phi)+delta_G(k_dion,k_phi))^2/4/(lambda+D(k_dion,k_phi)) +...
delta_el/2/pi*log(delta_el^2/((lambda+D(k_dion,k_phi)+delta_G(k_dion,k_phi))^2+delta_el^2)); % [eV] activation barrier, Eq.7
k(k_dion,k_phi) = k0*exp(-Ga(k_dion,k_phi)/(kB/e0*T)); % rate constant
j(k_dion,k_phi) = -2*k(k_dion,k_phi)*Nad*e0*(1-theta(k_dion,k_phi)); % [A/m^2] HER current density, Eq.8

  end

end

%% Plot results

color1 = [210 34 37]/255;
color2 = [16 118 63]/255;
color3 = [37 48 122]/255;

% Figure 5a, simulated current density
figure(1)
hold on
box on
h1 = plot(phi_range_RHE, j(1,:)/10, '-', 'LineWidth', 2, 'Color', color1);
h2 = plot(phi_range_RHE, j(2,:)/10, '-', 'LineWidth', 2, 'Color', color2);
h3 = plot(phi_range_RHE, j(3,:)/10, '-', 'LineWidth', 2, 'Color', color3);
xlim([-0.82, -0.2])
```

```
set(gca, 'XColor', [0 0 0], 'YColor', [0 0 0], 'FontSize', 12);
lgd = legend([h1 h2 h3], {'K^+', 'Na^+', 'Li^+'});
set(lgd, 'Box', 'off');

% Figure S2a, cation coverage
figure(2)
hold on
box on
h1 = plot(phi_range_RHE, theta(1,:), '-', 'LineWidth', 2, 'Color', color1);
h2 = plot(phi_range_RHE, theta(2,:), '-', 'LineWidth', 2, 'Color', color2);
h3 = plot(phi_range_RHE, theta(3,:), '-', 'LineWidth', 2, 'Color', color3);
xlim([-0.82, -0.2])
ylim([0 0.2])
set(gca, 'XColor', [0 0 0], 'YColor', [0 0 0], 'FontSize', 12);
lgd = legend([h1 h2 h3], {'K^+', 'Na^+', 'Li^+'});
set(lgd, 'Box', 'off');

% Figure S2c, OHP potential
figure(3)
hold on
box on
h1 = plot(phi_range_RHE, phi_OHP(1,:), '-', 'LineWidth', 2, 'Color', color1);
h2 = plot(phi_range_RHE, phi_OHP(2,:), '-', 'LineWidth', 2, 'Color', color2);
h3 = plot(phi_range_RHE, phi_OHP(3,:), '-', 'LineWidth', 2, 'Color', color3);
xlim([-0.82, -0.2])
set(gca, 'XColor', [0 0 0], 'YColor', [0 0 0], 'FontSize', 12);
lgd = legend([h1 h2 h3], {'K^+', 'Na^+', 'Li^+'});
set(lgd, 'Box', 'off');

% Figure 5e, Tafel slope for K and Li
figure(4)
hold on
box on
plot(phi_range_RHE(2:end), -0.01./diff(log10(-j(1,:)))*1000, '-', 'LineWidth', 2, 'Color', color1);
plot(phi_range_RHE(2:end), -0.01./diff(log10(-j(3,:)))*1000, '-', 'LineWidth', 2, 'Color', color3);
xlim([-0.82, -0.2])
set(gca, 'XColor', [0 0 0], 'YColor', [0 0 0], 'FontSize', 12);

% Figure S3b, Tafel slope for Na
figure(5)
hold on
box on
plot(phi_range_RHE(2:end), -0.01./diff(log10(-j(2,:)))*1000, '-', 'LineWidth', 2, 'Color', color2);
xlim([-0.82, -0.2])
set(gca, 'XColor', [0 0 0], 'YColor', [0 0 0], 'FontSize', 12);


%% Comparision with the model neglecting cation adsorption

for k_dion = 1:length(dion_list)

    dion = dion_list(k_dion); % [m] effective cation size
    delta2 = dion/2; % [m] thickness of outer Helmholtz layer (OHL)
    fsize = 2*cC0*1e3*(dion)^3*NA; % size factor

    for k_phi = 1:length(phi_range_RHE)
```

```
        phiM = phi_range_RHE(k_phi); % [V] electrode potential vs RHE

        % solve the EDL model
        EDL =  @(x)sign(x)*2*R*T/F*asinh(sqrt(1/2/fsize*(exp(fsize/2*(F*lD*x/R/T/epsb)^2)-1))) ...
           + x*(delta1/eps1+delta2/eps2)...
           -(phiM - phipzc -0.059*pH); % Eq.2
        x0 = [-0.1]; % initial guess
        lb = [-10]; % lower limit
        ub = [10]; % upper limit
        Res=lsqnonlin(EDL,x0,lb,ub); % nonlinear optimization
        sigmafree(k_dion,k_phi) = Res(1); % [C/m^2] free surface charge density
        E_OHL_wo(k_dion,k_phi) = -sigmafree(k_dion,k_phi)/eps2; % electric field strength without cation
adsorption
        phi_OHP(k_dion,k_phi) =
sign(sigmafree(k_dion,k_phi))*2*R*T/F*asinh(sqrt(1/2/fsize*(exp(fsize/2*(F*lD*sigmafree(k_dion,k_phi)/R/T/
epsb)^2)-1))); % OHP potential
        phi_IHP(k_dion,k_phi) = phi_OHP(k_dion,k_phi)+sigmafree(k_dion,k_phi)/eps2*delta2; % IHP potential
        delta_G(k_dion,k_phi) = phiM - 0.059*pH - E_eq - phi_OHP(k_dion,k_phi); % [eV] changes of Gibbs free
energy
        D_wo(k_dion,k_phi) = -B*E_OHL_wo(k_dion,k_phi) + D0; % [eV] bond strength, Eq. 6
        Ga(k_dion,k_phi) =
(lambda+D_wo(k_dion,k_phi)+delta_G(k_dion,k_phi))^2/4/(lambda+D_wo(k_dion,k_phi)) +...
        delta_el/2/pi*log(delta_el^2/((lambda+D_wo(k_dion,k_phi)+delta_G(k_dion,k_phi))^2+delta_el^2)); %
activation barrier, Eq.7
        k(k_dion,k_phi) = k0*exp(-Ga(k_dion,k_phi)/(kB/e0*T)); % rate constant
        j(k_dion,k_phi) = -2*k(k_dion,k_phi)*Nad*e0*(1-theta(k_dion,k_phi)); % HER current density, Eq 8

    end

end

% Figure S2b, H-OH bond strength
figure(6)
hold on
box on
h1 = plot(phi_range_RHE, D(1,:), '-',  'LineWidth', 2, 'Color', color1);
h2 = plot(phi_range_RHE, D(2,:), '-',  'LineWidth', 2, 'Color', color2);
h3 = plot(phi_range_RHE, D(3,:), '-',  'LineWidth', 2, 'Color', color3);
h4 = plot(phi_range_RHE, D_wo(1,:), '--','LineWidth', 2, 'Color', color1);
xlim([-0.82, -0.2])
set(gca, 'XColor', [0 0 0], 'YColor', [0 0 0], 'FontSize', 12);
lgd = legend([h1 h2 h3 h4], {'K^+', 'Na^+', 'Li^+', '\theta_{K^+}=0'});
set(lgd, 'Box', 'off');

% Figure 5e, Tafel slope for K and Li
figure(4)
hold on
box on
plot(phi_range_RHE(2:end), -0.01./diff(log10(-j(1,:)))*1000, '--', 'LineWidth', 2, 'Color', color1);
plot(phi_range_RHE(2:end), -0.01./diff(log10(-j(3,:)))*1000, '--', 'LineWidth', 2, 'Color', color3);
xlim([-0.82, -0.2])
set(gca, 'XColor', [0 0 0], 'YColor', [0 0 0], 'FontSize', 12);

% Figure S3b, Tafel slope for Na
figure(5)
hold on
box on
```

```
plot(phi_range_RHE(2:end), -0.01./diff(log10(-j(2,:)))*1000, '--', 'LineWidth', 2, 'Color', color2);
xlim([-0.82, -0.2])
set(gca, 'XColor', [0 0 0], 'YColor', [0 0 0], 'FontSize', 12);

% Figure 5c, electric field strength for K+
figure (7)
hold on
box on
fill([phi_range_RHE fliplr(phi_range_RHE)], [E_OHL(1,:) fliplr(E_OHL_wo(1,:))], [0.8 0.8 0.8], ...
   'FaceAlpha', 0.5, 'EdgeColor', 'none'); % fill the region between two lines
plot(phi_range_RHE, E_OHL(1,:), 'color',color1, 'LineWidth', 2);
plot(phi_range_RHE, E_OHL_wo(1,:),'--', 'color','k', 'LineWidth', 2);
xlim([-0.82, -0.2])
ylim([0.8e9 1.8e9])
set(gca, 'XColor', [0 0 0],'YColor', [0 0 0], 'FontSize', 12);

% Figure S3a, electric field strength for Na+
figure (8)
hold on
box on
fill([phi_range_RHE fliplr(phi_range_RHE)], [E_OHL(2,:) fliplr(E_OHL_wo(2,:))], [0.8 0.8 0.8], ...
   'FaceAlpha', 0.5, 'EdgeColor', 'none'); % fill the region between two lines
plot(phi_range_RHE, E_OHL(2,:), 'color',color2, 'LineWidth', 2);
plot(phi_range_RHE, E_OHL_wo(2,:),'--', 'color','k', 'LineWidth', 2);
xlim([-0.82, -0.2])
ylim([0.8e9 1.8e9])
set(gca, 'XColor', [0 0 0],'YColor', [0 0 0], 'FontSize', 12);

% Figure 5d, electric field strength for Li+
figure (9)
hold on
box on
fill([phi_range_RHE fliplr(phi_range_RHE)], [E_OHL(3,:) fliplr(E_OHL_wo(3,:))], [0.8 0.8 0.8], ...
   'FaceAlpha', 0.5, 'EdgeColor', 'none'); % fill the region between two lines
plot(phi_range_RHE, E_OHL(3,:), 'color',color3, 'LineWidth', 2);
plot(phi_range_RHE, E_OHL_wo(3,:), '--','color','k', 'LineWidth', 2);
xlim([-0.82, -0.2])
ylim([0.8e9 1.8e9])
set(gca, 'XColor', [0 0 0],'YColor', [0 0 0], 'FontSize', 12);
```

Fig. 6 and Fig. S4

```
%% Parameters
clc
clear all

% Experimental conditions
T  = 298; % [K] temperature
pH = 13; % solution pH
phiM = -0.6; % potential vs RHE

% Constant parameters
NA = 6.02e23; % [1/mol] Avogadro number
e0 = 1.6e-19; %[C] elementary charge
h = 6.626e-34; % [J.s] Planck constant
kB = 1.38e-23; %[J/K] Boltzmann constant
F = e0*NA; % [C/mol] Faraday constant
R = kB*NA; % [J/mol/K] gas constant
eps0 = 8.85e-12; %[F/m] vacuum permittivity

% EDL parameters
epsb = 78.5*eps0; % [F/m] bulk permittivity
delta1 = 2.0e-10; % [m] thickness of Inner Helmholtz layer (IHL)
eps1 = 8*eps0; % [F/m] permittivity of the IHL
eps2 = 30*eps0; % [F/m] permittivity of the OHL
phi_pzc = 0.5; % [V] potential of zero charge vs SHE
Nad = 1.4e19; % [m^-2] number density of active sites
Xi = 0.85; % charge number per adsorption cation
theta_max = 0.5; % maximum coverage
gamma = 3; % lateral interaction coefficient

% Kinetic parameters
lambda = 3; % [eV] reorganization energy
E_eq = -1.24; % [V] equilibrium potential of hydrogen adsorption
delta_el = 3; % [eV] electronic interaction strength
k0 = 1*kB*T/h; % [1/s] prefactor
B = 1.7e-9; % [e0.m] coefficient for electric field effects, Eq.6
D0 = 4.7; % [eV] field-free H-OH bond strength

%% Solve the model

dion_list = [6e-10 7e-10 8e-10]; % [m] effective cation size, [K, Na, Li]
Eref_list = [-1.19 -1.27 -1.65]; % [V] cation adsorption potential, [K, Na, Li]
concen_list = [0.105 0.125 0.15 0.2 0.35 0.6]; % [M] cation concentration


for k_dion = 1:length(dion_list)

  dion = dion_list(k_dion); % [m] effective cation size
  delta2 = dion/2; % [m] thickness of outer Helmholtz layer (OHL)
  E_ref = Eref_list(k_dion); % [V] cation adsorption potential

  for k_con = 1:length(concen_list)

    cC0 = concen_list(k_con); % [M] bulk cation concentration
    lD = sqrt(epsb*R*T/F^2/2/(cC0*1e3)); % [m] Debye length
```

```
        fsize = 2*cC0*1e3*(dion)^3*NA; % size factor

        % solve the cation adsorption coverage
        Coverage = @(x)log(x/(theta_max-x))+ gamma*x + (1-Xi)*e0*(phiM-0.059*pH-E_ref)/kB/T - log(cC0); %
Eq.4
        x0 = [0.1]; % initial guess
        lb = [0]; % lower limit
        ub = [theta_max]; % upper limit
        Res=lsqnonlin(Coverage,x0,lb,ub); % nonlinear optimization
        theta(k_dion,k_con) = Res(1); % cation coverage
        sigma_ad(k_dion,k_con) = e0*Nad*Xi*theta(k_dion,k_con); % surface charge contribution of adsored
cations

        % solve the EDL model
        EDL =  @(x)sign(x)*2*R*T/F*asinh(sqrt(1/2/fsize*(exp(fsize/2*(F*lD*x/R/T/epsb)^2)-1))) ...
           + x*(delta1/eps1+delta2/eps2)...
           - sigma_ad(k_dion,k_con)*delta1/eps1 ...
           -(phiM - phi_pzc -0.059*pH); % Eq.3
        x0 = [-0.1]; % initial guess
        lb = [-10]; % lower limit
        ub = [10]; % upper limit
        Res=lsqnonlin(EDL,x0,lb,ub); % nonlinear optimization
        sigmafree(k_dion,k_con) = Res(1); % [C/m^2] free surface charge density
        E_OHL(k_dion,k_con) = -sigmafree(k_dion,k_con)/eps2; % [V/m] electric field strength, Eq.5
        phi_OHP(k_dion,k_con) =
sign(sigmafree(k_dion,k_con))*2*R*T/F*asinh(sqrt(1/2/fsize*(exp(fsize/2*(F*lD*sigmafree(k_dion,k_con)/R/T
/epsb)^2)-1))); % OHP potential
        phi_IHP(k_dion,k_con) = phi_OHP(k_dion,k_con)+sigmafree(k_dion,k_con)/eps2*delta2; % IHP potential
        delta_G(k_dion,k_con) = phiM - 0.059*pH - E_eq - phi_OHP(k_dion,k_con); % [eV] changes of Gibbs free
energy, Eq.7
        D(k_dion,k_con) = -B*E_OHL(k_dion,k_con) + D0; % [eV] bond strength, Eq.6
        Ga(k_dion,k_con) = (lambda+D(k_dion,k_con)+delta_G(k_dion,k_con))^2/4/(lambda+D(k_dion,k_con)) +...
        delta_el/2/pi*log(delta_el^2/((lambda+D(k_dion,k_con)+delta_G(k_dion,k_con))^2+delta_el^2)); % [eV]
activation barrier, Eq.7
        k(k_dion,k_con) = k0*exp(-Ga(k_dion,k_con)/(kB/e0*T)); % rate constant
        j(k_dion,k_con) = -2*k(k_dion,k_con)*Nad*e0*(1-theta(k_dion,k_con)); % HER current density, Eq.8

    end

end

%% Plot results

color1 = [210 34 37]/255;
color2 = [16 118 63]/255;
color3 = [37 48 122]/255;
color4 = [168 30 50]/255;
color5 = [42 110 63]/255;
color6 = [22 24 35]/255;

% Figure 6a, simulated current density
figure(1)
hold on
box on
h1 = plot(log10(concen_list), log10(-j(1,:)/10), 'o','LineWidth',1,'color',color1,'MarkerEdgeColor',
color1,'MarkerFaceColor', color1,'MarkerSize', 8);
```

```
h2 = plot(log10(concen_list), log10(-j(2,:)/10), 'o','LineWidth',1,'color',color2,'MarkerEdgeColor',
color2,'MarkerFaceColor', color2,'MarkerSize', 8);
h3 = plot(log10(concen_list), log10(-j(3,:)/10), 'o','LineWidth',1,'color',color3,'MarkerEdgeColor',
color3,'MarkerFaceColor', color3,'MarkerSize', 8);
xlim([-1, -0.199])
ylim([-1.5, 0.5])
set(gca, 'XColor', [0 0 0], 'YColor', [0 0 0], 'FontSize', 12);
lgd = legend([h1 h2 h3], {'K^+', 'Na^+', 'Li^+'});
set(lgd, 'Box', 'off');

% Figure 6c, surface charge densities of K
figure(2)
hold on
box on
b = bar(log10(concen_list), [-sigmafree(1,:)*100; sigma_ad(1,:)*100 ], 'stacked');
b(1).FaceColor = color4;
b(2).FaceColor = color5;
set(b, 'BarWidth', 0.6);
plot(log10(concen_list), (-sigmafree(1,:)+sigma_ad(1,:))*100, '--o', 'LineWidth', 1.5,'color',color6,
'MarkerFaceColor', color6,'MarkerSize', 8);
ylim([0, 80])
set(gca, 'XColor', [0 0 0],'YColor', [0 0 0], 'FontSize', 12);

% Figure 6d, surface charge densities of Li
figure(3)
hold on
box on
b = bar(log10(concen_list), [-sigmafree(3,:)*100; sigma_ad(3,:)*100 ], 'stacked');
b(1).FaceColor = color4;
b(2).FaceColor = color5;
set(b, 'BarWidth', 0.6);
plot(log10(concen_list), (-sigmafree(3,:)+sigma_ad(3,:))*100, '--o', 'LineWidth', 1.5,'color',color6,
'MarkerFaceColor', color6,'MarkerSize', 8);
ylim([0, 80])
set(gca, 'XColor', [0 0 0],'YColor', [0 0 0], 'FontSize', 12);

% Figure 6e, OHP potential
figure(4)
hold on
box on
h1 = plot(log10(concen_list), phi_OHP(1,:), '-o','LineWidth',1,'color',color1,'MarkerEdgeColor',
color1,'MarkerFaceColor', color1,'MarkerSize', 8);
h2 = plot(log10(concen_list), phi_OHP(2,:), '-o','LineWidth',1,'color',color2,'MarkerEdgeColor',
color2,'MarkerFaceColor', color2,'MarkerSize', 8);
h3 = plot(log10(concen_list), phi_OHP(3,:), '-o','LineWidth',1,'color',color3,'MarkerEdgeColor',
color3,'MarkerFaceColor', color3,'MarkerSize', 8);
xlim([-1, -0.2])
ylim([-0.4, 0])
set(gca, 'XColor', [0 0 0], 'YColor', [0 0 0], 'FontSize', 12);
lgd = legend([h1 h2 h3], {'K^+', 'Na^+', 'Li^+'});
set(lgd, 'Box', 'off');

% Figure 6f, electric field strength
figure(5)
hold on
box on
```

```
h1 = plot(log10(concen_list), E_OHL(1,:), '-o','LineWidth',1,'color',color1,'MarkerEdgeColor',
color1,'MarkerFaceColor', color1,'MarkerSize', 8);
h2 = plot(log10(concen_list), E_OHL(2,:), '-o','LineWidth',1,'color',color2,'MarkerEdgeColor',
color2,'MarkerFaceColor', color2,'MarkerSize', 8);
h3 = plot(log10(concen_list), E_OHL(3,:), '-o','LineWidth',1,'color',color3,'MarkerEdgeColor',
color3,'MarkerFaceColor', color3,'MarkerSize', 8);
xlim([-1, -0.2])
ylim([4e8, 14e8])
set(gca, 'XColor', [0 0 0], 'YColor', [0 0 0], 'FontSize', 12);
lgd = legend([h1 h2 h3], {'K^+', 'Na^+', 'Li^+'});
set(lgd, 'Box', 'off');

% Figure S4a, cation adsorption coverage
figure(6)
hold on
box on
h1 = plot(log10(concen_list), theta(1,:), '-o','LineWidth',1,'color',color1,'MarkerEdgeColor',
color1,'MarkerFaceColor', color1,'MarkerSize', 8);
h2 = plot(log10(concen_list), theta(2,:), '-o','LineWidth',1,'color',color2,'MarkerEdgeColor',
color2,'MarkerFaceColor', color2,'MarkerSize', 8);
h3 = plot(log10(concen_list), theta(3,:), '-o','LineWidth',1,'color',color3,'MarkerEdgeColor',
color3,'MarkerFaceColor', color3,'MarkerSize', 8);
xlim([-1, -0.2])
ylim([0, 0.25])
set(gca, 'XColor', [0 0 0], 'YColor', [0 0 0], 'FontSize', 12);
lgd = legend([h1 h2 h3], {'K^+', 'Na^+', 'Li^+'});
set(lgd, 'Box', 'off');

% Figure S4b, surface charge densities of Na
figure(7)
hold on
box on
b = bar(log10(concen_list), [-sigmafree(2,:)*100; sigma_ad(2,:)*100 ], 'stacked');
b(1).FaceColor = color4;
b(2).FaceColor = color5;
set(b, 'BarWidth', 0.6);
plot(log10(concen_list), (-sigmafree(2,:)+sigma_ad(2,:))*100, '--o', 'LineWidth', 1.5,'color',color6,
'MarkerFaceColor', color6,'MarkerSize', 8);
ylim([0, 80])
set(gca, 'XColor', [0 0 0],'YColor', [0 0 0], 'FontSize', 12);
```

Fig. 7

```
%% Parameters

clc
clear all

% Experimental conditions
T  = 298; % [K] temperature
pH = 11; % solution pH
phiM = -0.6; % [V] potential vs RHE

% Constant parameters
NA = 6.02e23; % [1/mol] Avogadro number
e0 = 1.6e-19; %[C] elementary charge
h = 6.626e-34; % [J.s] Planck constant
kB = 1.38e-23; %[J/K] Boltzmann constant
F = e0*NA; % [C/mol] Faraday constant
R = kB*NA; % [J/mol/K] gas constant
eps0 = 8.85e-12; %[F/m] vacuum permittivity

% EDL parameters
epsb = 78.5*eps0; % [F/m] bulk permittivity
delta1 = 2.0e-10; % [m] thickness of Inner Helmholtz layer (IHL)
eps1 = 8*eps0; % [F/m] permittivity of the IHL
dion = 6e-10; % [m] effective cation size
delta2 = dion/2; % [m] thickness of outer Helmholtz layer (OHL)
eps2 = 30*eps0; % [F/m] permittivity of the OHL
phi_pzc = 0.5; % [V] potential of zero charge vs SHE
Nad = 1.4e19; % m^-2, number density of active sites
Xi = 0.85; % charge number per adsorption cation
theta_max = 0.5; % maximum coverage
gamma = 3; % lateral interaction coefficient

% Kinetic parameters
lambda = 3; % [eV] reorganization energy
E_eq = -1.24; % [V] equilibrium adsorption potential of hydrogen
delta_el = 3; % [eV] electronic interaction strength
k0 = 1*kB*T/h; % [1/s] prefactor
B = 1.7e-9; % [e0.m] coefficient for electric field effects, Eq.6
D0 = 4.7; % [eV] field-free H-OH bond strength

%% Solve the model

Eref_list = [-1.5 -1.2 -0.9]; % [V] cation adsorption potential vs SHE
concen_list = [0.006 0.026 0.051 0.101]; % [M] cation concentration list

for k_Eref = 1:length(Eref_list)

   E_ref = Eref_list(k_Eref); % cation adsorption potential

   for k_con = 1:length(concen_list)

      cC0 = concen_list(k_con); % [M] bulk cation concentration
      lD = sqrt(epsb*R*T/F^2/2/(cC0*1e3)); % [m] Debye length
      fsize = 2*cC0*1e3*(dion)^3*NA; % size factor
```

```
        % solve the cation adsorption coverage
        Coverage = @(x)log(x/(theta_max-x))+ gamma*x + (1-Xi)*e0*(phiM-0.059*pH-E_ref)/kB/T - log(cC0); %
Eq.4
        x0 = [0.1]; % initial guess
        lb = [0]; % lower limit
        ub = [theta_max]; % upper limit
        Res=lsqnonlin(Coverage,x0,lb,ub); % nonlinear optimization
        theta(k_Eref,k_con) = Res(1); % cation coverage
        sigma_ad(k_Eref,k_con) = e0*Nad*Xi*theta(k_Eref,k_con); % surface charge contribution of adsored
cations

        % solve the EDL model
        EDL =  @(x)sign(x)*2*R*T/F*asinh(sqrt(1/2/fsize*(exp(fsize/2*(F*lD*x/R/T/epsb)^2)-1))) ...
           + x*(delta1/eps1+delta2/eps2)...
           - sigma_ad(k_Eref,k_con)*delta1/eps1 ...
           -(phiM - phi_pzc -0.059*pH); % Eq.3
        x0 = [-0.1]; % initial guess
        lb = [-10]; % lower limit
        ub = [10]; % upper limit
        Res=lsqnonlin(EDL,x0,lb,ub); % nonlinear optimization
        sigmafree(k_Eref,k_con) = Res(1); % [C/m^2] free surface charge density
        E_OHL(k_Eref,k_con) = -sigmafree(k_Eref,k_con)/eps2; % [V/m] electric field strength, Eq.5
        phi_OHP(k_Eref,k_con) =
sign(sigmafree(k_Eref,k_con))*2*R*T/F*asinh(sqrt(1/2/fsize*(exp(fsize/2*(F*lD*sigmafree(k_Eref,k_con)/R/T/
epsb)^2)-1))); % OHP potential
        phi_IHP(k_Eref,k_con) = phi_OHP(k_Eref,k_con)+sigmafree(k_Eref,k_con)/eps2*delta2; % IHP potential
        delta_G(k_Eref,k_con) = phiM - 0.059*pH - E_eq - phi_OHP(k_Eref,k_con); % [eV] changes of Gibbs free
energy, Eq.7
        D(k_Eref,k_con) = -B*E_OHL(k_Eref,k_con) + D0; % [eV] bond strength, Eq.6
        Ga(k_Eref,k_con) = (lambda+D(k_Eref,k_con)+delta_G(k_Eref,k_con))^2/4/(lambda+D(k_Eref,k_con)) +...
        delta_el/2/pi*log(delta_el^2/((lambda+D(k_Eref,k_con)+delta_G(k_Eref,k_con))^2+delta_el^2)); % [eV]
activation barrier, Eq.7
        k(k_Eref,k_con) = k0*exp(-Ga(k_Eref,k_con)/(kB/e0*T)); % rate constant
        j(k_Eref,k_con) = -2*k(k_Eref,k_con)*Nad*e0*(1-theta(k_Eref,k_con)); % [A/m^2] HER current density,
Eq.8

    end

end

%% Plot results

color1 = [168 30 50]/255;
color2 = [42 110 63]/255;
color3 = [22 24 35]/255;

% experimental data, mA/cm^2
j_Au_11 = [-4.4 -4 -3.7 -3.5]+3; % exp, Au, pH=11, -0.6V
j_Pt_9 = [-2.99 -2.83 -2.75 -3.53]+3;% exp, Pt, pH=9, -0.3V
j_Pt_11 = [-2.79 -3.24 -3.61 -3.87]+3; % exp, Pt, pH=11, -0.4V

% Figure 7a, experiments
figure(1)
hold on;box on
plot(log10(concen_list),j_Au_11,'s','LineWidth',2,'color',color1,'MarkerEdgeColor', color1,'MarkerSize', 8);
xlim([-2.5 -0.8])
```

```
ylim([-1.5 0.5])
set(gca, 'XColor', [0 0 0],'YColor', [0 0 0], 'FontSize', 11);

figure(2)
hold on;box on
plot(log10(concen_list),j_Pt_9,'s','LineWidth',2,'color',color2,'MarkerEdgeColor', color2,'MarkerSize', 8);
xlim([-2.5 -0.8])
ylim([-1.0 1])
set(gca, 'XColor', [0 0 0],'YColor', [0 0 0], 'FontSize', 11);

figure(3)
hold on;box on
plot(log10(concen_list),j_Pt_11,'s','LineWidth',2,'color',color3,'MarkerEdgeColor', color3,'MarkerSize', 8);
xlim([-2.5 -0.8])
ylim([-1.0 1.0])
set(gca, 'XColor', [0 0 0],'YColor', [0 0 0], 'FontSize', 11);

% Figure 7b, simulations
figure(4)
hold on;box on
plot(log10(concen_list),log10(-j(1,:)/10),'o','LineWidth',2,'color',color1,'MarkerEdgeColor',
color1,'MarkerFaceColor', color1,'MarkerSize', 8);
xlim([-2.5 -0.8])
ylim([-1.0 0.5])
set(gca, 'XColor', [0 0 0],'YColor', [0 0 0], 'FontSize', 11);

figure(5)
hold on;box on
plot(log10(concen_list),log10(-j(2,:)/10),'o','LineWidth',2,'color',color2,'MarkerEdgeColor',
color2,'MarkerFaceColor', color2,'MarkerSize', 8);
xlim([-2.5 -0.8])
ylim([-1.0 0.0])
set(gca, 'XColor', [0 0 0],'YColor', [0 0 0], 'FontSize', 11);

figure(6)
hold on;box on
plot(log10(concen_list),log10(-j(3,:)/10),'o','LineWidth',2,'color',color3,'MarkerEdgeColor',
color3,'MarkerFaceColor', color3,'MarkerSize', 8);
xlim([-2.5 -0.8])
ylim([-2 -0.5])
set(gca, 'XColor', [0 0 0],'YColor', [0 0 0], 'FontSize', 11);
```
